# The middle Cambrian Linyi Lagerstätte from the North China Craton: a new window on Cambrian evolutionary fauna

**DOI:** 10.1093/nsr/nwac069

**Published:** 2022-04-05

**Authors:** Zhixin Sun, Fangchen Zhao, Han Zeng, Cui Luo, Heyo Van Iten, Maoyan Zhu

**Affiliations:** State Key Laboratory of Palaeobiology and Stratigraphy, Nanjing Institute of Geology and Palaeontology, and Center for Excellence in Life and Palaeoenvironment, Chinese Academy of Sciences, Nanjing 210008, China; College of Earth and Planetary Sciences, University of Chinese Academy of Sciences, Beijing 100049, China; State Key Laboratory of Palaeobiology and Stratigraphy, Nanjing Institute of Geology and Palaeontology, and Center for Excellence in Life and Palaeoenvironment, Chinese Academy of Sciences, Nanjing 210008, China; College of Earth and Planetary Sciences, University of Chinese Academy of Sciences, Beijing 100049, China; State Key Laboratory of Palaeobiology and Stratigraphy, Nanjing Institute of Geology and Palaeontology, and Center for Excellence in Life and Palaeoenvironment, Chinese Academy of Sciences, Nanjing 210008, China; State Key Laboratory of Palaeobiology and Stratigraphy, Nanjing Institute of Geology and Palaeontology, and Center for Excellence in Life and Palaeoenvironment, Chinese Academy of Sciences, Nanjing 210008, China; Department of Geology, Hanover College, Hanover, IN 47243, USA; Department of Invertebrate Paleontology, Cincinnati Museum Center, Cincinnati, OH 45203, USA; State Key Laboratory of Palaeobiology and Stratigraphy, Nanjing Institute of Geology and Palaeontology, and Center for Excellence in Life and Palaeoenvironment, Chinese Academy of Sciences, Nanjing 210008, China; College of Earth and Planetary Sciences, University of Chinese Academy of Sciences, Beijing 100049, China

**Keywords:** Cambrian explosion, exceptional preservation, North China Craton, Mollisoniidae, Zhangxia Formation, Drumian

## Abstract

The rapid appearance of major animal groups and complex marine communities during the Cambrian explosion is recorded in large part in Burgess Shale-type lagerstätten. However, the restricted temporal and spatial distribution of known lagerstätten continues to hinder the formation of a comprehensive perspective on Cambrian evolutionary faunas. Here we describe the Linyi Lagerstätte (ca. 504 mya), a new Cambrian Miaolingian lagerstätte from the Zhangxia Formation in Shandong Province, North China. The Linyi Lagerstätte contains a variety of well-preserved soft-bodied fossils, among which the non-trilobite arthropods, particularly the mollisoniids and radiodonts, are the most important groups. The new assemblage is remarkable for its excellent preservation of arthropod limbs, eyes and guts, as well as for its close similarity in taxonomic composition to Laurentian lagerstätten. The distinctive Linyi Lagerstätte holds great promise for providing additional insights into the morphological disparity, community structure and paleogeographic range of marine faunas during the middle Cambrian (Miaolingian).

## Teaser

The large tempo-spatial discrepancy of distribution of the soft-bodied fossil lagerstätten limits our understanding of the Cambrian explosion of animals on Earth. The discovery of the Linyin Lagerstätte (∼504 mya) in North China reported by Sun et al. provides a new window into this critical evolutionary event.

## INTRODUCTION

**Figure 1.**
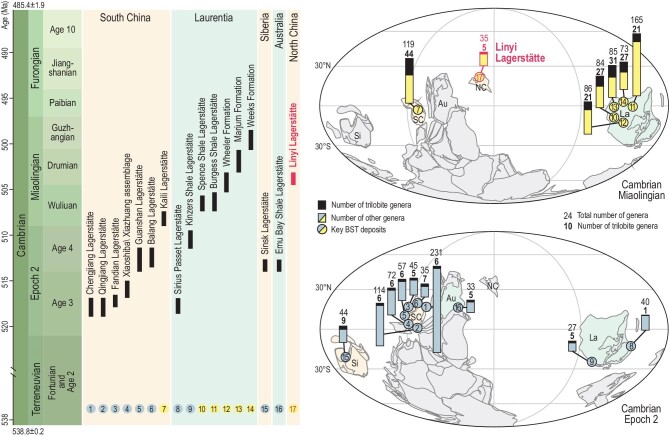
Spatial and temporal distribution and taxonomic diversity of 16 major Cambrian lagerstätten, and the position of the Linyi Lagerstätte. Numbers in the stratigraphic distribution chart correspond to those plotted in the paleogeographic map. The diversity data for the key Cambrian lagerstätten dealt with in this study were obtained from Refs [7,8,12,13,49] and this study. Global paleogeography for the early and middle Cambrian are modified from Ref. [50] and Ref. [41]. Abbreviations: SC, South China; La, Laurentia; Si, Siberia; Au, Australia; NC, North China.

Current understanding of the origin and early diversification of metazoans and marine communities during the Cambrian explosion is based primarily on the exceptional fossil records of Burgess Shale-type (BST) lagerstätten. Many fossils from these sites contain relic soft parts, which are normally absent in the fossil record, and thus provide critical evidence bearing on the structure of Cambrian marine communities and the appearance of key evolutionary innovations [1–3]. Together, nearly 20 Cambrian lagerstätten preserve a detailed record of early animal evolution from Cambrian Epoch 2 to the Miaolingian [4,5], and new lagerstätten are still being discovered [6–9]. Nevertheless, most of the well-known Cambrian lagerstätten are restricted to a few terranes, with most of them (over a dozen) occurring in South China (Epoch 2) or Laurentia (Miaolingian) (Fig. 1). This striking geographical imbalance is particularly evident in the middle Cambrian, with the major Miaolingian lagerstätten, including the Burgess Shale in British Columbia (western Canada) and five lagerstätten in the Great Basin (western USA), being located predominantly in Laurentia [10–13]. The Miaolingian period witnessed the apogee of the Cambrian evolutionary fauna and was the key interval between the aftermath of the Cambrian explosion and the Great Ordovician Biodiversification Event [2,14,15]. However, the scarcity and limited spatial distribution of Miaolingian lagerstätten have hindered efforts to understand the evolutionary and ecological changes that occurred during this time. Hence the need for an intensified search for Miaolingian lagerstätten in other terranes such as North China, where recent discoveries of typical Miaolingian BST fossils [16–18] have made this area a particularly inviting prospect.

**Figure 2.**
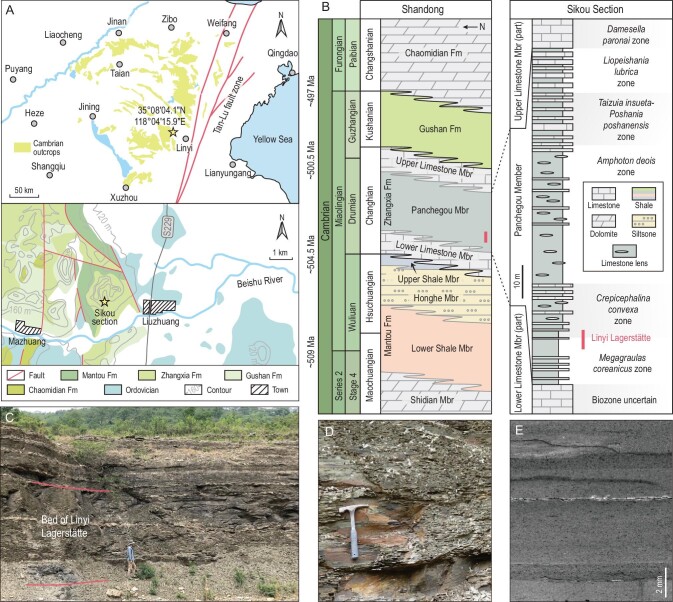
Maps showing the paleogeography, stratigraphy and location of the Linyi Lagerstätte. (A) Regional map of eastern North China, showing the locations of Cambrian outcrops, and geological map of the study area, showing the extent of Cambrian outcrops and the location of the Sikou section. (B) Stratigraphic correlation between Shandong Province and the global scale, and stratigraphic column with fossil horizons of the Sikou section. (C) Photograph of the Sikou section, showing the beds of the Linyi Lagerstätte in outcrop, taken facing south; person for scale is 1.9 m tall. (D) Close-up of (C), with the length of the hammer being 28 cm. (E) Polished slab showing multiple background (dark) and event (light) beds. Abbreviations: Fm, Formation; Mbr, Member.

Here we report the discovery of a new Miaolingian lagerstätte in the lower part of the Panchegou Member of the Zhangxia Formation (lower Drumian) in Shandong Province, North China (Figs 1 and 2). The Linyi Lagerstätte contains a diverse and well-preserved BST fossil assemblage, providing unique insights into the structure, taphonomy and paleobiogeographical relationships of Miaolingian marine communities outside of North America.

## RESULTS

The Linyi Lagerstätte is exposed in the Sikou section (35°08^′^04.1^″^N, 118°04^′^15.9^″^E) on the western side of Liuzhuang village, a northwestern suburb of Linyi City (Fig. 2A and B). BST fossils were collected from alternating event and background beds in a 5-meter-thick interval of black and greenish shale (Fig. 2C–E) situated 10.5–15.2 m above the base of the Panchegou Member of the Zhangxia Formation. This horizon has yielded over 35 fossil taxa, including four trilobites, one agnostoid, at least nine soft-bodied arthropods, two lophophorates, at least seven sponges, one chancelloriid, one priapulid, seven problematica, four macroalgae and four trace fossils (Supplementary Fig. 5). Arthropods are the most diverse metazoan group, comprising 12 species and two indeterminate taxa. The Linyi Lagerstätte ranges from the upper part of the *Megagraulas coreanicus* trilobite Zone to the lower part of the *Crepicephalina convexa* trilobite Zone [19], an interval corresponding to the early Drumian [19,20]. It is slightly younger than the Burgess Shale and is equivalent in age to the Wheeler Formation Lagerstätte (Fig. 1). The Panchegou Member was deposited in a platform margin to outer shelf setting and contains scattered carbonate platform deposits [21].

The presence in the Linyi Lagerstätte of taxa occupying diverse ecological niches points to the existence of a complex food web (Supplementary Figs 5B and 7). As in the Chengjiang biota [22], the fossil community of the Linyi biota was dominated by epifaunal organisms (trilobites, chelicerate arthropods, lophophorates and sponges), though nektonic arthropods (radiodonts and bivalved arthropods) also seem to have played important roles. Predators account for ∼25% of the species, a proportion similar to that of the Burgess Shale [23] but slightly less than that of the Chengjiang biota.

### Arthropods

**Figure 3.**
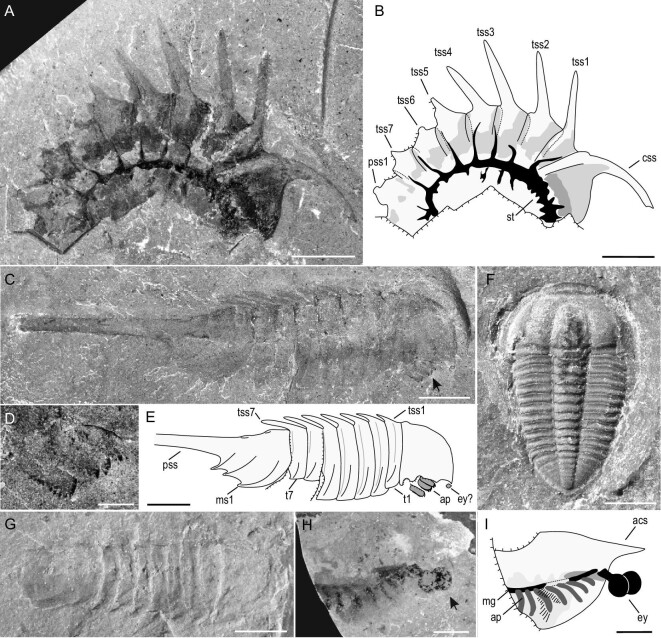
Euarthropods from the Linyi Lagerstätte. (A) *Thelxiope spinosa* (NIGP 176314). (B) Interpretative drawing of (A). (C) *Thelxiope tangi* sp. nov., the holotype (NIGP 176315); arrow indicates the limbs. (D) Close-up of the limbs in (C), showing the cephalic gnathobases. (E) Interpretative drawing of (C). (F) Complete exoskeleton of the trilobite *Changqingia puteata*, showing the digestive structure (NIGP 176316). (G) *Mollisonia symmetrica* (NIGP 176317). (H) *Isoxys shandongensis* with appendages and a pair of eyes (NIGP 176318). (I) Interpretative drawing of (H). Abbreviations: acs, anterior cardinal spine; ap, appendages; css, cephalic sagittal spine; ey, eyes; mg, midgut; ms, marginal spine; pss, pygidial sagittal spines; t1–7, thoracic tergites; st, soft tissue; tss, thoracic sagittal spine. Scale bars: 5 mm (A–D), 2 mm (G–I), 1 mm (E, F).

Trilobites, the most common arthropods, are represented by four species in four genera (Fig. 3F, Supplementary Figs 1 and 2F). Hundreds of complete trilobites have been collected, and some of them show exceptional preservation of portions of the digestive system (Fig. 3F and Supplementary Fig. 1B) [24]. The less abundant agnostoids are represented exclusively by *Ammagnostus laiwuwnsis* (Supplementary Fig. 2E).

The chelicerates are represented by two mollisoniid genera, *Thelxiope* and *Mollisonia*. *Thelxiope* is a poorly known arthropod characterized by well-developed spines on the dorsal exoskeleton. Previously, this rare genus comprised four species collectively represented by fewer than 10 specimens from Laurentia and West Gondwana [25]. The most distinctive species, *T*. *spinosa*, is represented by a single specimen from the Wheeler Formation of Utah [25,26]. The single known specimen of *T*. *spinosa* from Sikou (Fig. 3A and B) represents the first occurrence of this species outside of Laurentia, and it preserves obvious soft tissue. *T*. *tangi* sp. nov. from the Linyi Lagerstätte preserves cephalic gnathobases with teeth that are differentiated antero-posteriorly (Fig. 3C–E and Supplementary Fig. 4, also see Supplementary Data). The discovery of *Thelxiope* in the Linyi Lagerstätte expands the known diversity, disparity and palaeogeographical distribution of this rare arthropod. The complete specimen of *Mollisonia* from Sikou can be assigned to the type species *M. symmetrica* (Fig. 3G), and it represents the first discovery of this genus in North China. Isolated gnathobases of chelicerate-like taxa have also been found in Linyi (Supplementary Fig. 2C and D). These specimens display homogeneous teeth on the masticatory margin, an arrangement that is different from that of *Habelia* and *Sidneyia* from the Burgess Shale and from that of *Wisangocaris* from the Emu Bay Shale.

**Figure 4.**
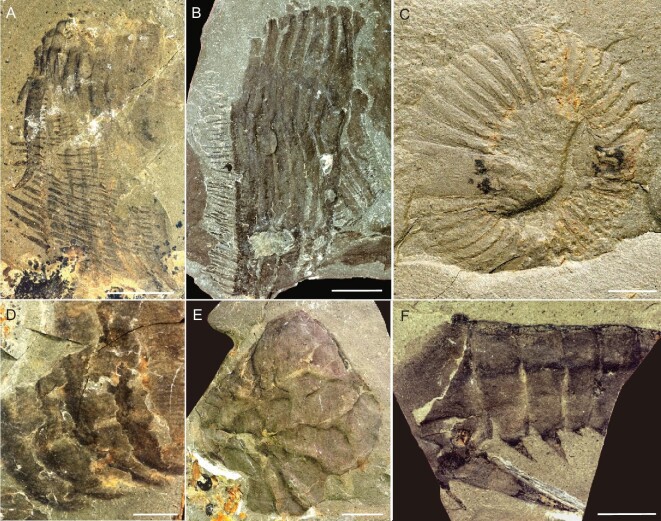
Radiodonts from the Linyi Lagerstätte. (A–E) Body parts of *Cordaticaris striatus*. (A and B) Frontal appendages (NIGP 176319, 176320). (C) Oral cone (NIGP 173116). (D) Set of setal structures (NIGP 173114). (E) Smaller central element (NIGP 173112). (F) Frontal appendage of an amplectobeluid (NIGP 176321). Scale bars: 2 mm (C, D), 5 mm (A, E, F), 10 mm (B).

Radiodonts are also common. The hurdiid *Cordaticaris striatus*, the most common non-trilobite arthropod in the Linyi Lagerstätte, exhibits a heart-shaped central element and frontal appendages equipped with at least seven extremely long blade-like endites (Fig. 4A–E). This species is thought to be intermediate in feeding mode between pure sediment sifters and suspension feeders [18]. Two frontal appendages of an amplectobeluid (Fig. 4F and Supplementary Fig. 2I) have not yet been assigned to a genus. These two fossils increase the known disparity and geographic range of the amplectobeluids, and make North China the third region outside of South China and Laurentia where this group has been found [26].

Also present are the cosmopolitan bivalved arthropods *Isoxys* and *Tuzoia*. The commonest of these is *I*. *shandongensis*, which was the first species to be described from the Linyi Lagerstätte [27]; one specimen of this arthropod preserves appendages and a pair of eyes (Fig. 3H and I). *Tuzoia* is represented by an incomplete specimen of *T*. cf. *manchuriensis* (Supplementary Fig. 3I). Finally, a single specimen of an incomplete trunk bearing reniform digestive glands with ramified diverticula (Supplementary Fig. 2A and B), together with another, incomplete arthropod with at least 10 segments, suggest the presence of additional new arthropod taxa in the Linyi Lagerstätte (Supplementary Fig. 3C).

### Other groups

**Figure 5.**
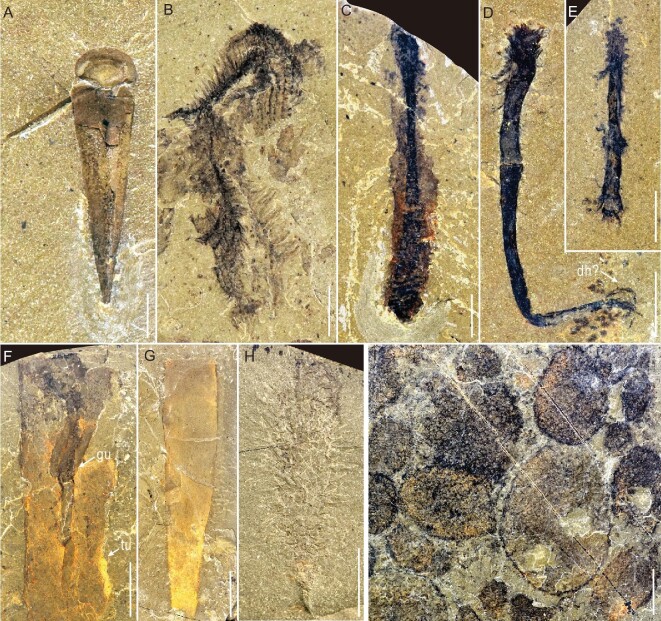
Representative fossils from the Linyi Lagerstätte. (A) *Novakotheca weifangensis* (NIGP 176324). (B) Worm-like animal D (NIGP 176333). (C) Worm-like animal A (NIGP 176330). (D and E) Worm-like animal B, showing the possible discoidal holdfast (dh?) (NIGP 176334, 176335). (F) *Selkirkia* sp., showing the coiled gut (gu) and tube (tu) (NIGP 176322). (G) Tube of *Selkirkia* sp. (NIGP 176323). (H) ?*Allonnia* (NIGP 176326). (I) Monospecific cluster of the sponge *Diagoniella* sp. (NIGP 176328). Scale bars: 2 mm (A–E), 5 mm (F–H), 10 mm (I).

Animals other than arthropods are also abundant in the Linyi Lagerstätte, with sponges being the second most diverse group. Some of the sponges are here provisionally assigned to the genera *Diagoniella*, *Halichondrites*, *Hydnodictya* and ?*Palaeosaccus*, one new protospongiid genus, one indeterminate reticulosan and one possible aspicular sponge (Fig. 5I and Supplementary Fig. 3H). *Diagoniella* is present in many other Miaolingian lagerstätten, but the two indeterminate protospongiid sponges may represent new taxa.

Chancelloriids are (Fig. 5H) represented by major sclerites resembling those of *Allonnia*, but there are also sclerites possibly bearing four main rays like those in *Archiasterella*. Therefore, we provisionally identify the chancelloriids as ?*Allonnia*.

Possible *Selkirkia* tubes are not rare, but only a few specimens preserve relic soft parts (Fig. 5F and G). The large tubes have nearly parallel sides and are somewhat similar to *S. spencei* from the Spence Shale. Also, the anterior coiled gut in some specimens is similar to that of *Paraselkirkia* from the Chengjiang biota. Still, until a definitive proboscis is found the identity of these tubes will remain uncertain.

Brachiopods and hyoliths make up 67% of the total number of collected specimens, with brachiopods alone making up 50% of the total (Supplementary Fig. 5A). However, the diversity of these two taxa is very low, with just one species in each group having been identified thus far (Supplementary Fig. 3E). Some specimens of the hyolith *Novakotheca weifangensis* preserve relic soft tissue including the helens and digestive tract (Fig. 5A and Supplementary Figs 2G and 3J).

Problematica are represented by medusiform (Supplementary Fig. 3F) and vermiform animals. Some of the vermiform fossils bear seriated transverse rings and an obvious digestive system (Fig. 5C and Supplementary Figs 2H and 3A), while others display a well-defined long pedicle and discoidal holdfast (Fig. 5D and E). Some display a posterior structure (Supplementary Fig. 3B) reminiscent of that of archaic hemichordates such as *Gyaltsenglossus* and *Spartobranchus* from the Burgess Shale. A single specimen (Fig. 5B) bearing several rows of long bristles can be interpreted as an unknown polychaete. Exactly how many species all of the foregoing incomplete specimens represent is unknown at present.

Algae and trace fossils are abundant and occur in most beds (Supplementary Fig. 2K) [28]. Some hyolith-bearing coprolites (Supplementary Fig. 3G) are strikingly similar to elliptical aggregates described from the Chengjiang biota; these structures may have been produced by epibenthic predators [29].

### Taphonomy

Like other typical BST lagerstätten [30,31], fossiliferous strata in the Linyi Lagerstätte consist of stacked couplets of thin event and background mudstone beds (Fig. 2E). The 1-mm-thick background beds yield abundant but poorly preserved shelly fossils, mostly trilobites, brachiopods and hyolithids. In contrast, the 1- to 10-mm-thick event beds contain exquisitely preserved soft-bodied organisms.

The Linyi fossils are preserved as dark-colored carbonaceous films (Supplementary Fig. 6), a major taphonomic pathway shared with other BST deposits worldwide [32]. In addition to enrichment in carbon, elemental mapping has also revealed the presence of iron in a reddish-colored worm specimen (Supplementary Fig. 6C). Micrographs showed that the iron was derived from the oxidation of pyrite. The preservational fidelity of typical BST fossils is high and includes the eye in *Isoxys* (Fig. 3H), limbs and digestive system in *Thelxiope* and the ‘great appendage’ arthropod (Fig. 3A–E, and Supplementary Fig. 2A–D), and the gut in *Changqingia* (Fig. 3F), *Maotunia* (Supplementary Fig. 1B and figures in [29]), *Novakotheca* (Fig. 5A and Supplementary Figs 2G and 3J), *Thelxiope spinosa* (Fig. 3A) and *Selkirkia* (Fig. 5F). In short, the well-preserved fossils of the Linyi Lagerstätte promise to yield new anatomical data relating to the early evolution of animals.

## DISCUSSION

Current understanding of metazoan diversity and evolutionary faunas following the Cambrian explosion is based primarily on localities in South China and Laurentia, especially the Chengjiang and Burgess Shale lagerstätten. To be sure, Cambrian soft-bodied fossils have been found in other places [33,34], but only the Emu Bay Shale (Stage 4) in East Gondwana and the Sinsk Formation in Siberia (Stage 4) exhibit a preservational fidelity and taxonomic diversity [35,36] comparable to those of the lagerstätten of South China and Laurentia (Fig. 1). This uneven spatial distribution is especially apparent in the middle Cambrian: BST fossils are available for most of the Miaolingian period, but with the exception of the Kaili biota in South China (earliest Wiliuan) [37], Miaolingian lagerstätten are known exclusively from the Spence Shale Member, Burgess Shale, and Wheeler, Marjum and Weeks formations in North America [10–13,26] (Fig. 1).

**Figure 6.**
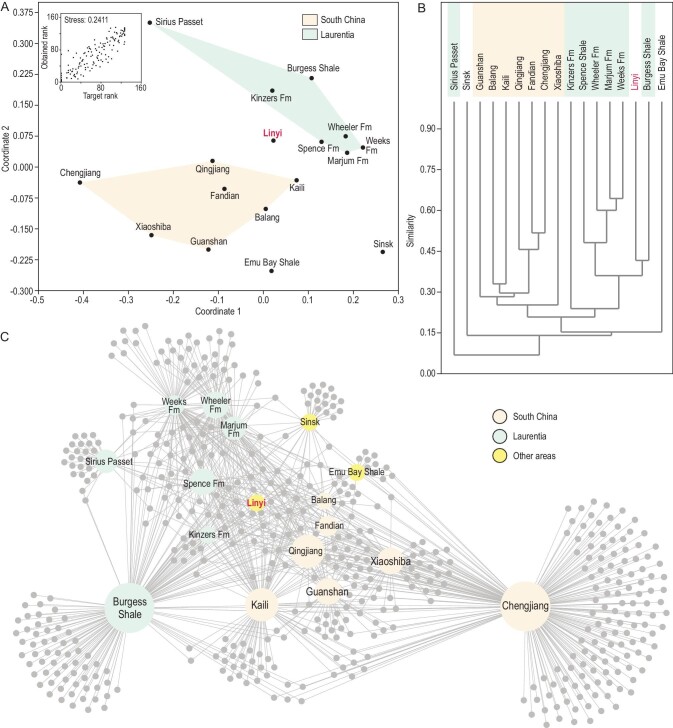
Biogeographic comparisons of Cambrian lagerstätten and the position of the Linyi Lagerstätte, based on the binary presence/absence data for 508 genera from 16 major Cambrian lagerstätten and the Linyi Lagerstätte. (A) Ordination plot of non-metric multidimensional scaling (nMDS) analysis (Raup & Crick similarity, analysis in PAST 4.03), stress = 0.2411. (B) Cluster analysis (Kulczynski similarity, analysis in PAST 4.03), CPCC = 0.869. (C) Bipartite network analysis (analysis in Gephi 0.9.2), size of the dots not to scale. Database (Supplementary Data 1) from Holmes *et al*. [49], Lerosey-Aubril *et al*. [12], Fu *et al*. [7] and Du *et al*. [8], with trilobites not included.

The limited known spatial distribution of Cambrian lagerstätten thus underscores the importance of the newly discovered Miaolingian Linyi Lagerstätte, which provides unique insights into the evolution and biogeography of Cambrian marine life. Several congeneric and even conspecific fossils are shared between the Linyi Lagerstätte and contemporaneous lagerstätte in Laurentia. Some of these taxa have been interpreted as cosmopolitan [38] or persistent taxa [39], while others, especially *Thelxiope* and *Mollisonia*, are rare even at their type localities [25]. The occurrence of *T. spinosa* and *M. symmetrica* in the Linyi Lagerstätte, and of *Sidneyia* and *Cambroraster* in the Upper Shale Member of the Mantou Formation [16,17], suggest a close relationship between the Miaolingian deposits of North China and those of Laurentia. This hypothesis is further supported by the results of cluster analysis, which show the Linyi Lagerstätte nested within the Laurentia group, next to the Burgess Shale (Fig. 6B). However, given the obvious differences between the Cambrian shelly faunas of North China and Laurentia [40], it remains unclear whether this pattern has any paleogeographic significance. Indeed, it is possible that middle Cambrian soft-bodied taxa exhibited no distinct biogeographic divisions, but further evaluation of this hypothesis will require additional data from other geographic units. Importantly, non-metric multidimensional scaling (nMDS) and network analyses of the major Cambrian lagerstätten suggest that the Linyi Lagerstätte may have provided a biogeographic link between East Gondwana and Laurentia (Fig. 6A and C), a hypothesis that is further bolstered by recent paleomagnetic data [41]. However, it is unclear whether the Linyi Lagerstätte was situated adjacent to Laurentia or represents an independent transitional region between Laurentia and Gondwana.

The BST succession in North China consists of the Lower Shale Member BST assemblage (upper Cambrian Stage 4 to lower Wuliuan) [42–44] and the Upper Shale Member BST assemblage (uppermost Wuliuan) in the Mantou Formation [16,17,45–48], and the Linyi Lagerstätte in the Panchegou Member (lower and middle Drumian) (Fig. 2B). Thus, North China is now an important region for investigating the early evolution of middle Cambrian animals, and its Miaolingian deposits have great potential for yielding additional exceptional biotas. Since the discovery of the Chengjiang biota in 1984, South China has gradually become the principal area for the study of early Cambrian lagerstätte. The discovery of the Linyi Lagerstätte may also open a new chapter in the study of middle Cambrian BST deposits in North China.

## MATERIALS AND METHODS

### Geological setting and specimens

The middle Miaolingian (*c.* Drumian) Zhangxia (Changhia) Formation is widely distributed in North China. In eastern Shandong Province it conformably overlies the early Miaolingian Mantou (Manto) Formation and consists of three members: the Lower Limestone Member, the Linyi Lagerstätte-bearing Panchegou Member and the Upper Limestone Member. Unlike the other two members, which are dominated by oolitic and algal limestone, the Panchegou Member is composed mainly of shale [21]. It crops out only in eastern Shandong Province (though probably in southeastern Liaoning Province as well) and is thought to have been deposited in a platform margin to outer shelf environment with scattered carbonate platform deposits [21]. At least five trilobite zones have been recognized in the Panchegou Member, from the lowermost *Megagraulas coreanicus* Zone to the uppermost *Liopeishania lubrica* Zone, which together correspond to most of the Drumian [21] (Fig. 2B).

Fossils *(n* = 3023) were collected or counted in the Sikou section in localized excavations of four beds averaging 30 cm in thickness and 3 m² in area. All specimens are housed in the Nanjing Institute of Geology and Palaeontology, Chinese Academy of Sciences (NIGP).

### Methods

Photographs were taken under crossed-polarized light using a Nikon D810 camera fitted with a Nikon AF-S Nikkor 105 mm lens. Back scattered electron (BSE) image capture and energy dispersive spectrometer (EDS) analysis were performed using a TESCAN MAIA 3 GMU field-emission scanning electron microscope. Images were processed using Adobe Photoshop to adjust tone, contrast and brightness. Sedimentological observations were performed on polished slabs.

## Supplementary Material

nwac069_Supplemental_Files
