# Supplementary material for: The middle Cambrian Linyi Lagerstätte from the North China Craton: a new window on Cambrian evolutionary fauna
Source: Natl Sci Rev. 2022 Apr 5;9(7):nwac069. doi: 10.1093/nsr/nwac069 (PMC9273334; doi:10.1093/nsr/nwac069)
Supplement: nwac069_Supplemental_Files [file nwac069_supplemental_files.zip › Supplement_Text_and_Figures.pdf]

Supplementary Materials for

The middle Cambrian Linyi Lagerstätte from the North China Craton: a new window on the Cambrian evolutionary fauna

Zhixin Sun, Fangchen Zhao, Han Zeng, Cui Luo, Heyo Van Iten, Maoyan Zhu

Correspondence to: fczhao@nigpas.ac.cn (F. Z)

This PDF file includes:

Supplementary Text

Supplementary Figs. 1 to 7

References

Other Supplementary Materials for this manuscript include the following:

Supplementary Data 1 (Data for Figure 6: The binary presence/absence data including 508 genera from 16 major Cambrian lagerstätten and Linyi Lagerstätte, with trilobites not included.)

Supplementary Text

SYSTEMATIC PALAEONTOLOGY

Phylum EUARTHROPODA Lankester, 1904 (see Ortega-Hernández, 2016)
Class CHELICERATA? Heymons, 1901
Order MOLLISONIIDA Lerosey-Aubril et al., 2020a
Family MOLLISONIIDAE Lerosey-Aubril et al., 2020a
Genus THELXIOPE Simonetta and Delle Cave, 1975

Type species. *Thelxiope palaeothalassia* Simonetta and Delle Cave, 1975
Other included species. *Thelxiope holmani* Lerosey-Aubril et al., 2020b; *T. spinosa* (Conway Morris and Robison, 1988); *T. tangi* sp. nov. (this study); and *Thelxiope* sp. nov. A (Van Roy et al., 2010) in Lerosey-Aubril et al., 2020b.
Occurrences. The Wuliuan Burgess Shale Formation in British Columbia, Canada; the Drumian Wheeler Formation in the House Range, Utah, USA; The Drumian Zhangxia Formation in Shandong Province, North China; Tremadocian strata of the Fezouata Shale in south-eastern Morocco.

Thelxiope tangi sp. nov.
Fig. 3C–E and Supplementary Fig. 4

ZooBank LSID. Urn:lsid:zoobank.org:act:7F887DE4-767A-4E83-8F56-79DDDDA8087D.
Etymology. In honour of Yonggang Tang, a private fossil collector who provided invaluable assistance in our field work and research.
Diagnosis. Species of *Thelxiope* with narrow-based, posterodorsally projecting thoracic sagittal spines, straight tergopleural tips and spines, and a hypertrophied posteriormost pygidial sagittal spine.
Holotype. A laterally compressed complete exoskeleton, including part and counterpart, NIGP 176315 (Fig. 3C–E and Supplementary Fig. 4A–D).
Additional materials. Two pygidia, NIGP 176352, 176353 (Supplementary Fig. 4E, F).
Type locality and horizon. Sikou section in Linyi County, Shandong Province, North China. Lower part of the Panchegou Member, Zhangxia Formation, Cambrian Miaolingian, Drumian Stage (Changhian, *Megagraulas coreanicus* Zone)
Description. Length of the cephalic shield less than one fifth the total length (sag.) of the main body (excluding the spines), tergopleural regions broken (Supplementary Fig. 4C). Posterior margin straight, with the rounded bases of the broken sagittal spine still present on the dorsal side. Three mutually overlapping gnathobases present underneath the broken cephalic shield (Fig. 4D); masticatory margin exhibits more than ten homogeneous teeth, with the teeth gradually decreasing in size proximally.

Thorax constitutes about one half of the total main body length (13mm), composed of seven, mutually similar, articulated spinose tergites (T1–7); anterior part of each thoracic tergite concealed beneath the tergite immediately in front of it. Tergopleurae with rounded tips on the anterior margin and straight tips on the posterior margin, lateral margins subparallel to the sagittal axis. Tergopleural ridge posteriorly curved distally and reaching the lateral

margin close to its meeting point with the posterior margin. Sagittal spine slender and pointed, projecting posteriorly from the dorsal part of each tergite, increasing in length toward the pygidium. T1 notably narrower (tr.) than the cephalic shield and T2, owing to its shorter (tr.) tergopleural extent. Tergites nearly equal in length (sag.) along the entire thorax, but slightly increasing in width (tr.) from T2 to T4, then moderately decreasing in width in the posterior tergites.

Pygidium slightly longer (7.5mm; sag.) than the cephalic shield, representing almost one third of the total length (sag.) of the main body (Supplementary Fig. 4F). Three pairs of marginal spines separated by concave segments along the posterolateral margin, and three pairs of curved ridges running from the vicinity of the marginal spines toward the axial region. Three slender sagittal spines (pss1–3) project dorsally and increasingly posteriorly, with the first two pygidial sagittal spines (pss1–2) being indistinct and short. Hypertrophied third pygidial sagittal spine (pss3) long and straight, its length (sag.) exceeding three times the length of the pygidium; pss3 particularly robust, rate of taper small, narrowing gradually and distally to reach half its proximal diameter at the preserved distal tip.

Remarks. Morphological features such as similarly sized cephalic and pygidial shields, thorax composed of seven freely articulating tergites, and 11 well-developed sagittal spines on the dorsal exoskeleton, support the assignment of the new specimens to the genus *Thelxiope*. *T. tangi* is most similar to the type species, *T. palaeothalassia*: both have a hypertrophied, posteriormost pygidial sagittal spine as well as seven short, narrow-based thoracic sagittal spines. Nevertheless, the straight tergopleural tips and posterodorsally projecting thoracic sagittal spines in *T. tangi* make the two species easy to distinguish from one another. In these two characters, *T. tangi* is similar to *T. holmani*, but *T. tangi* differs from *T. holmani* in having a hypertrophied pygidial sagittal spine and narrow-based thoracic spines. Although the characteristics of the cephalic sagittal spine are not clear, *T. tangi* differs from *T. spinosa* in the shape of the thoracic sagittal spines. Therefore, the combination of narrow-based, posterodorsally projecting thoracic sagittal spines, straight tergopleural tips, and a hypertrophied posteriormost pygidial sagittal spine make *T. tangi* distinguishable from all other species in *Thelxiope*.

Thelxiope spinosa (Conway Morris and Robison, 1988)
Fig. 3A and B
1988 *Ecnomocaris spinosa* Conway Morris and Robison, pp. 27–30: fig. 19, 20.
1991 *Ecnomocaris spinosa* Conway Morris and Robison, p. 86: fig. 7.
2020 *Thelxiope spinosa* (Conway Morris and Robison, 1988); Lerosey-Aubril et al 2020b, p. 12, 14–17: fig. 5, 6.
Type locality and horizon. Upper Wheeler Formation (Miaolingian, Drumian) at the type locality (‘U-Dig quarry’ now) in the House Range, Millard County, Utah.
Additional material. A laterally compressed complete exoskeleton, NIGP 176314 (Fig. 3Aand B).
Description. Length of the cephalic shield about one fifth of the total length (sag.) of the main body (excluding the spines) when oriented laterally. Cephalic sagittal spine bent and long (css), broad-based, its length slightly exceeding the length (sag.) of the cephalon, obviously projecting anteriorly. Length of the thorax exceeds half of the total length (sag.) of the main body, thorax composed of seven, mutually similar, articulated spinose tergites (T1–7); anterior part of each thoracic tergite concealed beneath the tergite immediately in front of it. Sagittal spine (tss1–7) long and slender, approximately equal in length to the sagittal length of the corresponding thoracic tergite,

projecting dorsally and posteriorly from the dorsal posterior half (sag.) of each tergite. Morphology of the tergopleural tips unknown. Pygidium incomplete, preserved part shows a stout first sagittal spine (pss1).

Digestive-tract-like soft tissue preserved as a carbonaceous film extending throughout most of the body; soft tissue composed of a large axial band (st in Fig. 3B) and seven lateral extensions corresponding to the thoracic tergites.

Remark. Although the pygidial features of NIGP 176314 cannot be observed, the long, narrow-based thoracic spines and hypertrophied cephalic spine support the assignment of this specimen to *T. spinosa* (Conway Morris and Robison, 1988), which was originally described from the Wheeler Formation of Utah, USA. These same two features also distinguish the new specimen from other *Thelxiope*. The new specimen exhibits an obviously more curved and shorter cephalic sagittal spine compared with the type specimen, but this is not sufficient for assigning the new specimen to a new species of this genus. Since the cephalic shields of the new and type specimens are compressed in different postures (oblique versus lateral orientations), and the anterior most portion of the cephalic spine in the new specimen is not completely preserved, the observed differences in the anatomy of the cephalic spine may be attributable to taphonomic deformation or individual differences.

Supplementary Figs. 1-7

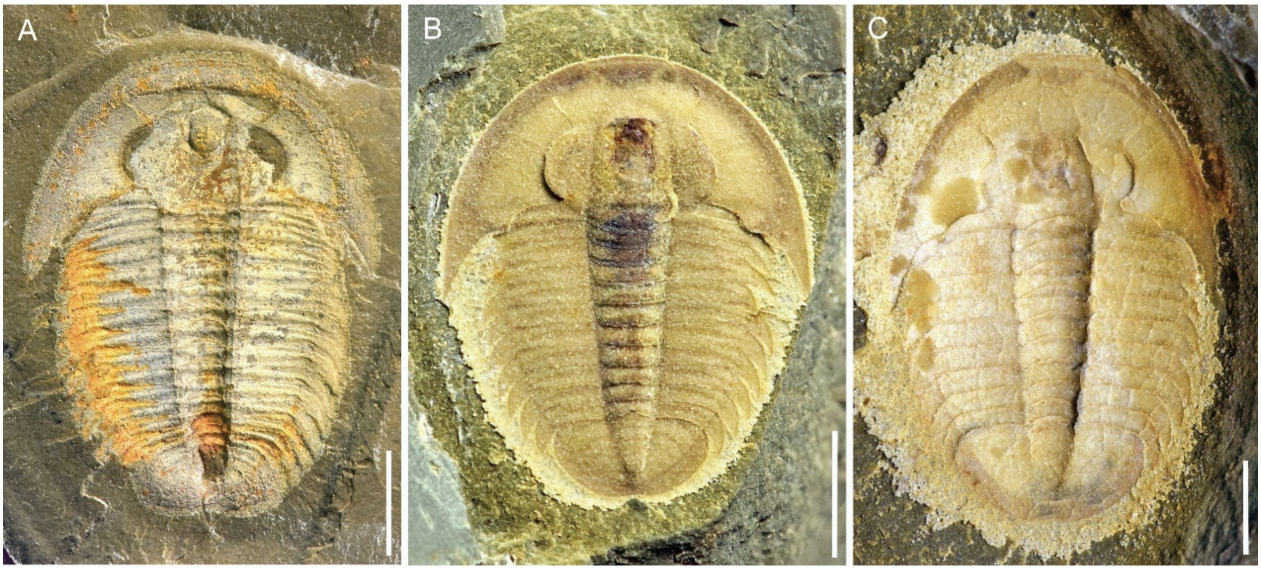

Supplementary Fig. 1. Trilobites from the Linyi Lagerstätte.

(A) *Eymekops transversa*, Yuan et al., 2012, NIGP 176340. (B) *Maotunia iddingsi* (Walcott, 1911), NIGP 176341. (C) *Szeaspis conicus*, Yuan et al., 2012, NIGP 176342. Scale bars, 5 mm (A) and 2 mm (B, C).

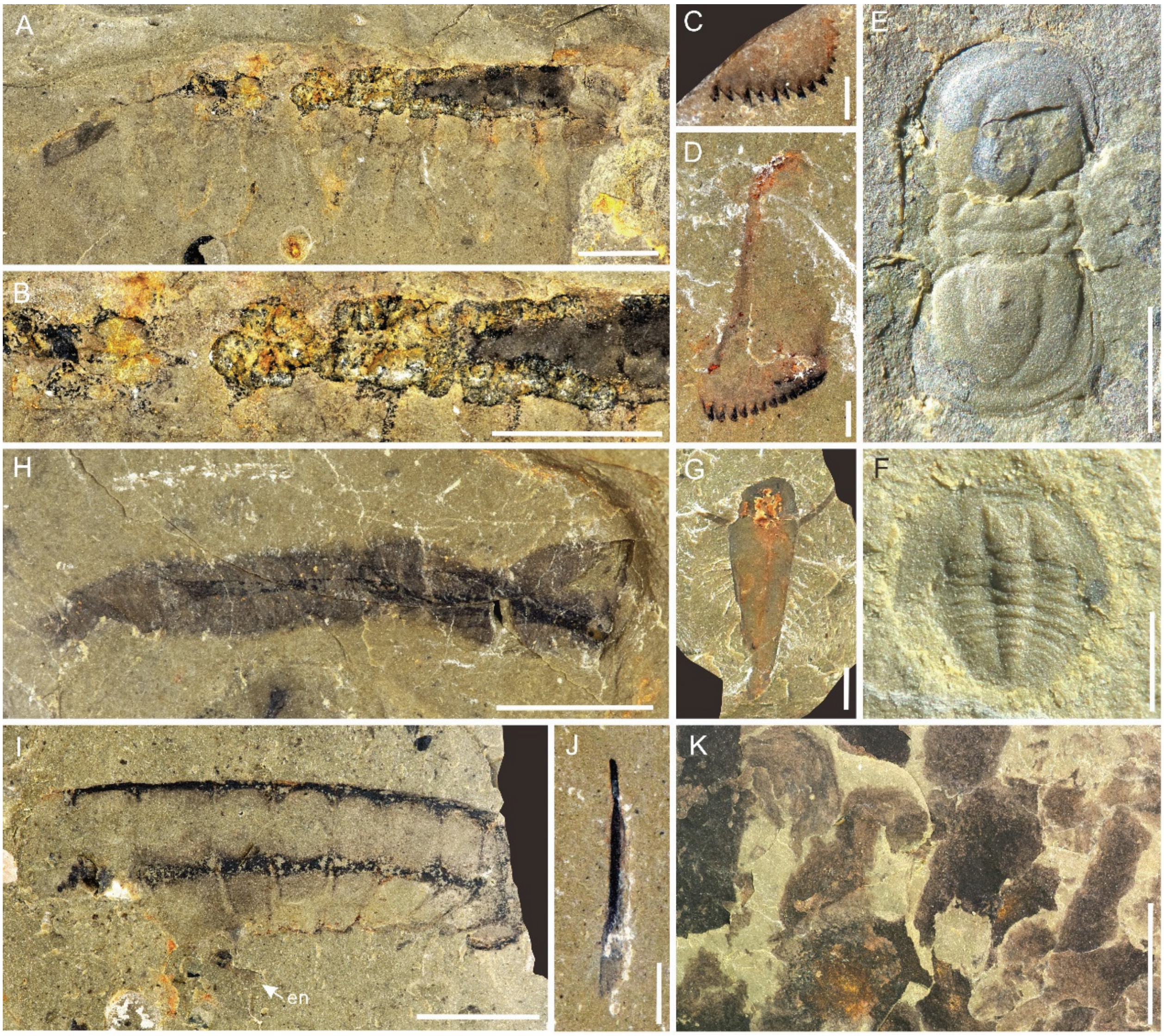

Supplementary Fig. 2. Typical fossils from the Linyi Lagerstätte.

(A) Undetermined 'great appendage' arthropod (NIGP 176343). (B) Close-up of (A), showing the reniform digestive glands with ramified diverticula. (C and D) Isolated gnathobases of *Thelxiope* (NIGP 176344). (E) Complete exoskeleton of the agnostid *Ammagnostus laiwuwnsis* (NIGP 176345). (F) Meraspides of the trilobite *Maotunia iddingsi* (NIGP 176346). (G) *Novakotheca weifangensis* with helens and gut (NIGP 176347). (H) Worm-like animal B (NIGP 176348). (I) Frontal appendage of amplectobeluid radiodont (NIGP 176349). (J) Worm-like animals C (NIGP 176350). (K) Undetermined banded algae (NIGP 176351). Abbreviation: en, endite. Scale bars, 1 mm (F), 2 mm (C–E, G, J), 5 mm (A, B, H, I) and 10 mm (K).

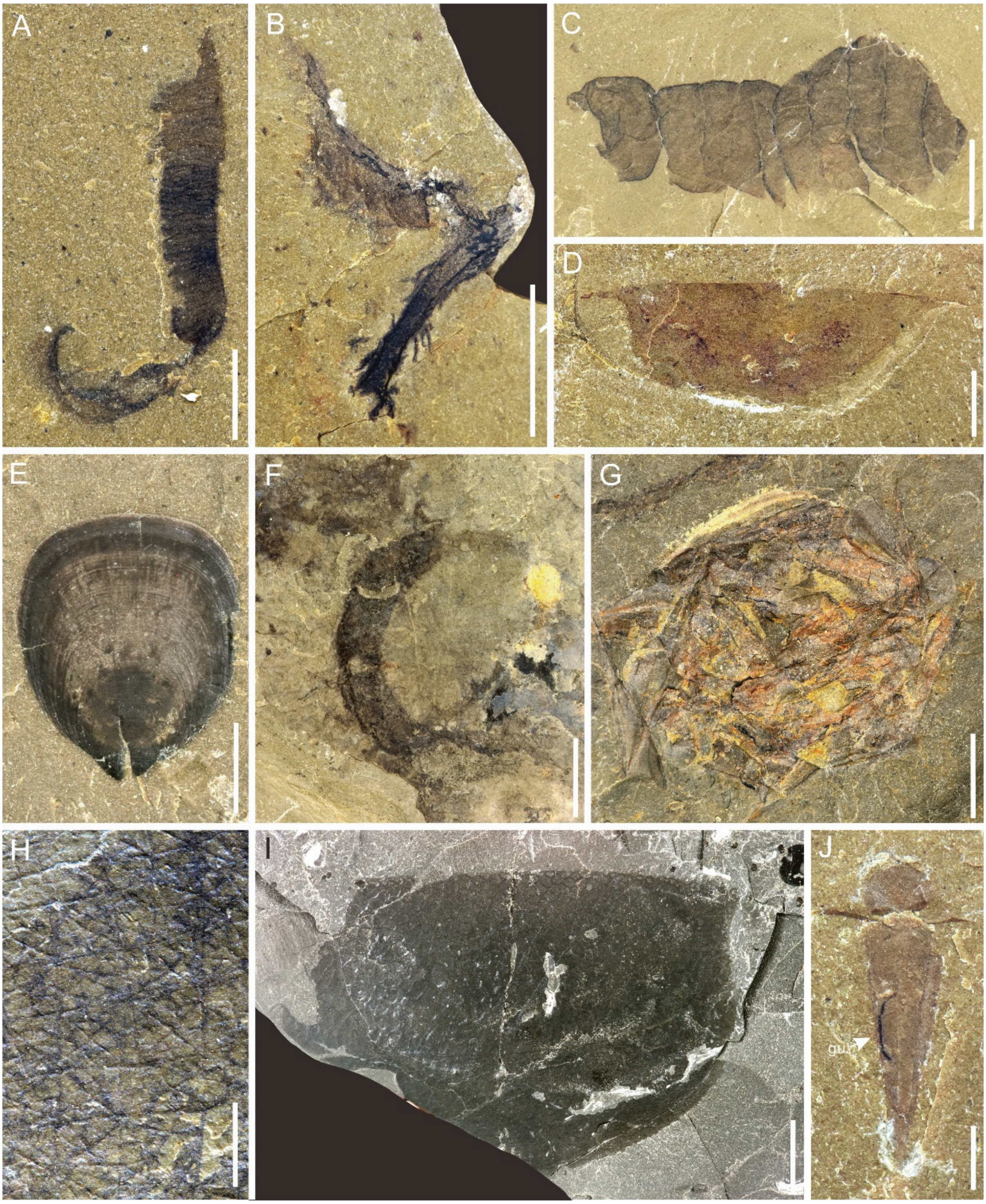

Supplementary Fig. 3. Typical fossils from the Linyi Lagerstätte.

(A) Worm-like animal A (NIGP 176332). (B) Indeterminate yunnanozoon-like animal (NIGP 176337). (C) An undetermined arthropod (NIGP 176339). (D) *Isoxys shandongensis* (NIGP 176292). (E) *Lingulella* sp. (NIGP 176327). (F) Medusiform ?*Eldonia* (NIGP 176336). (G) Ellipsoid coprolite containing hyoliths (NIGP 176338). (H) Partial enlarged view of a new protospongiid (NIGP 176329). (I) *Tuzoia* cf. *manchuriensis* (NIGP 176293). (J) *Novakotheca weifangensis*, showing the helens and gut (gu) (NIGP 176325). Scale bars, 2 mm (A, D, E, H, J), 5 mm (B, C, F, G) and 10 mm (I).

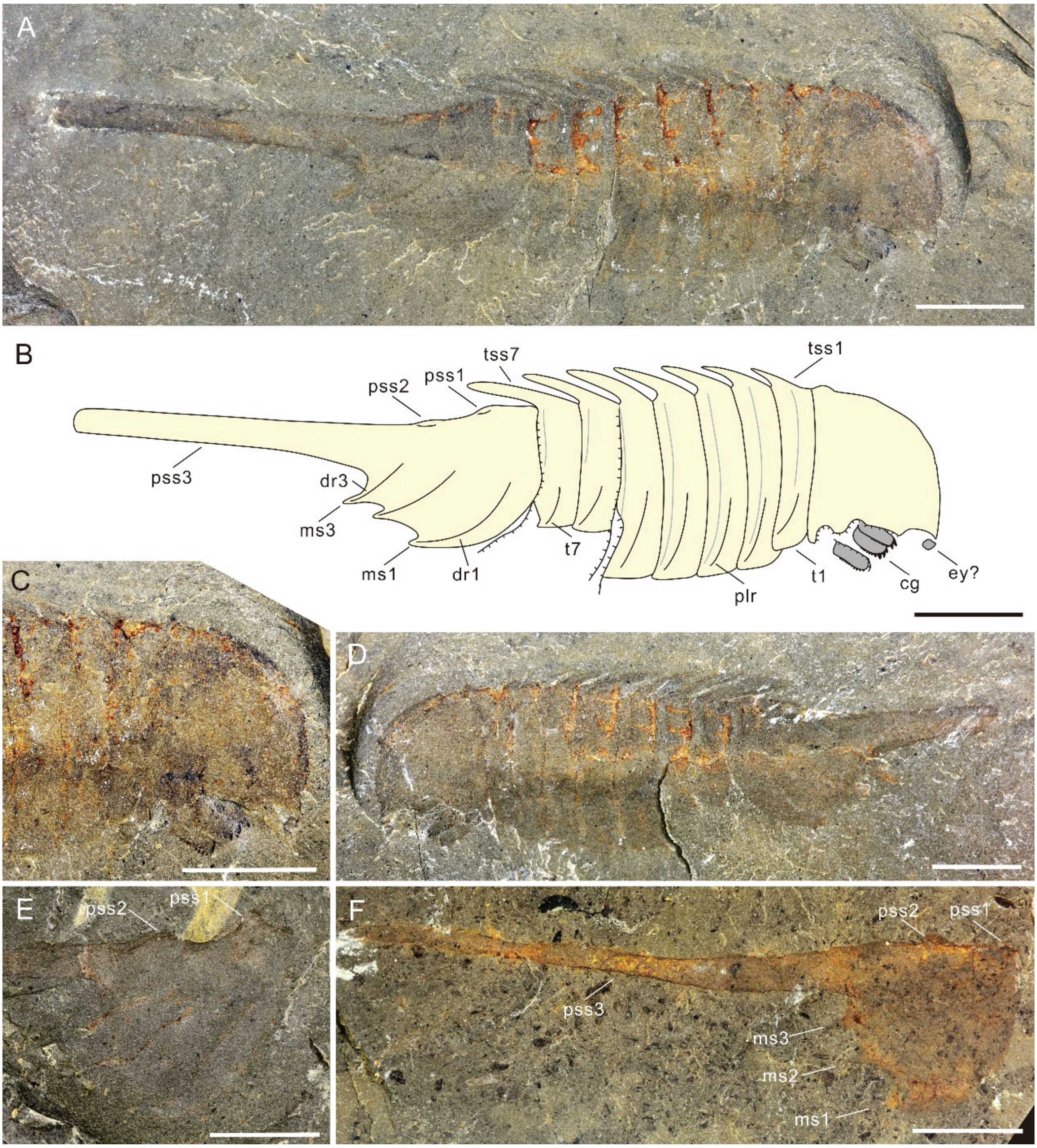

Supplementary Fig. 4. *Thelxiope tangi* sp. nov. from the Linyi Lagerstätte.

(A–D) Holotype specimen NIGP 176315. (A) Overall view of specimen; (B) Interpretative drawing of (A); (C) Close-up of (A); (D) Counter part of (A); (E), (F) Pygidia of *T. tangi*. NIGP 176352, 176353. Abbreviations: cg, cephalic gnathobases; dr, dorsal ridge; ey, eye; ms, marginal spine; plr, tergopleural ridge; pss, pygidial sagittal spine; t, thoracic tergite; tss, thoracic sagittal spine. Scale bars, 5 mm.

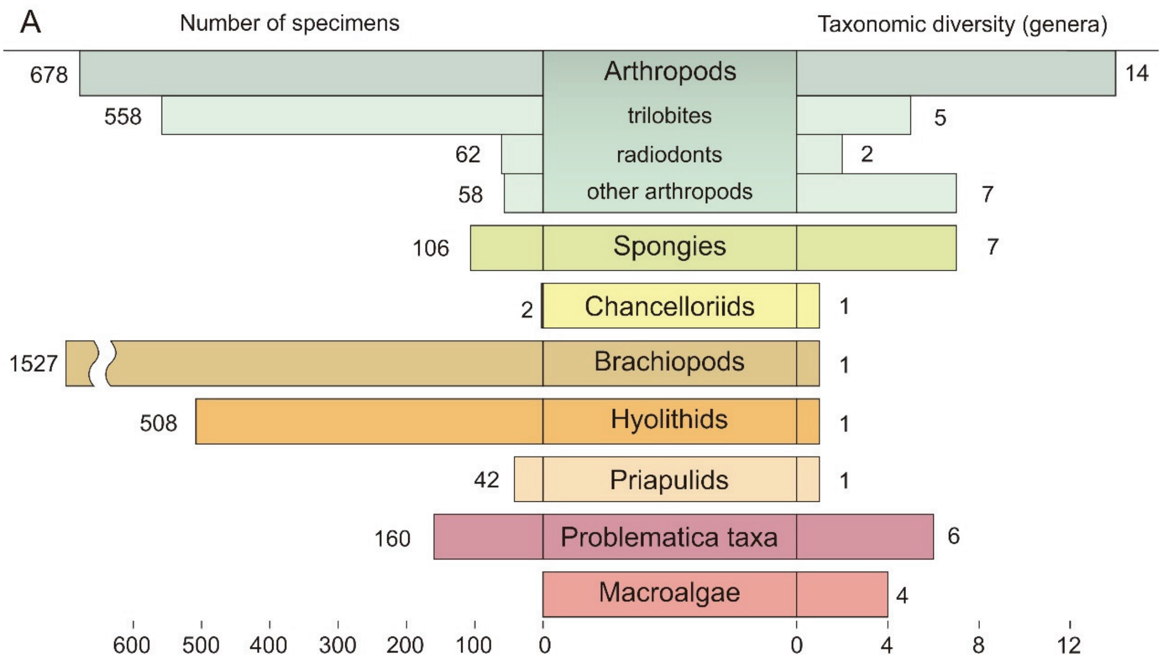

B Species	Groups	Ecology	No.	Species	Groups	Ecology	No.
Ammagnostus laiwuwnsis	Arthropods	NKDE	40	Indete protospongiid A	Spongies	ESSU	9
Maotunia iddingsi	Arthropods	EVOM	456	Indete protospongiid B	Spongies	ESSU	1
Szeaspis conicus	Arthropods	EVOM	2	non-spicular sponge	Spongies	ESSU	9
Changqingia puteata	Arthropods	EVOM	8	*?Allonnia*	Chancelloriids	ESHS	2
Eymekops transversa	Arthropods	EVOM	52	*Lingulella* sp.	Brachiopods	ESSU	1527
Cordaticaris striatus	Arthropods	NKSU	60	*Novakotheca weifangensis*	Hyolithids	EVOM	508
amplectobeluid sp.	Arthropods	NKHS	2	*Selkirkia* sp.	Priapulids	ISHS	42
Isoxys shandongensis	Arthropods	PEHS	48	worm-like animal A	undetermined	?EVUN	122
Tuzoia manchuriensis	Arthropods	PEHS	1	worm-like animal B	undetermined	?ESUN	18
Thelxiope tangi	Arthropods	EVHS	5	worm-like animal C	undetermined	?EVUN	13
Thelxiope spinosa	Arthropods	EVHS	1	worm-like animal D	undetermined	UNUN	1
Mollisonia symmetrica	Arthropods	EVHS	1	worm-like animal E	undetermined	UNUN	1
great appendage arthropods	Arthropods	EVHS	1	medusiform eldoniids	undetermined	PESU	3
Indete arthropods	Arthropods	?EVHS	1	*Fuxianospira gyrata*	Macroalgae	ES–	–
Diagoniella pera	Spongies	ESSU	73	*Sinocylindra yunnanensis*	Macroalgae	ES–	–
Hydnodictya sp.	Spongies	ESSU	5	*Morania fragmenta*	Macroalgae	ES–	–
Halichondrites sp.	Spongies	ESSU	8	Indete banded algae	Macroalgae	ES–	–
Indete hexactinellid sponge	Spongies	ESSU	1	35 species	8 groups	3023 specimens	

Supplementary Fig. 5. Taxonomic diversity and specimen abundance of the Linyi Lagerstätte, excluding trace fossils.

(A) Histogram showing the diversity and abundance for the major taxonomic groups. (B) Data matrix showing the biotic composition at the species level. Abbreviations for life habits: infaunal sessile (IS), epifaunal sessile (ES), epifaunal vagrant (EV), nektobenthic (NK), pelagic (PE), unknown (UN); Abbreviations for feeding strategy: suspension (SU), deposit (DE), hunter/scavenger (HS), omnivorous (OM), unknown (UN). Trilobite *Maotunia iddingsi* was counted using only complete individuals, and the number of microalgae were not included in the statistical analysis.

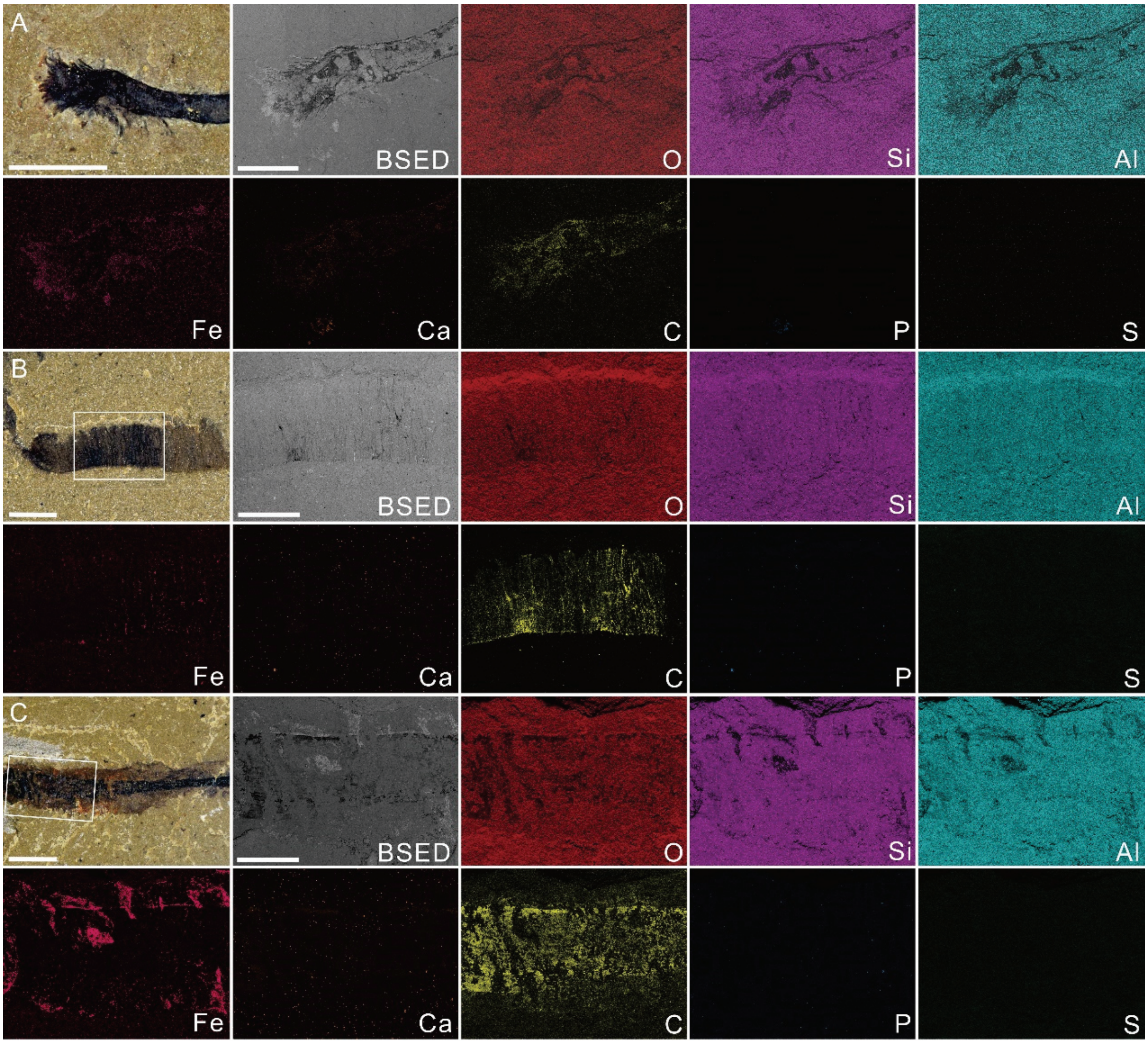

Supplementary Fig. 6. Elemental map analyses in the Linyi Lagerstätte, showing soft-bodied tissues preserved as carbonaceous films. (A) NIGP 176334. (B) NIGP 176332. (C) NIGP 176330. Abbreviations: BSED, Back-scattered Electron Detector. Scale bars: 0.5 mm in BSED and elemental maps, 2 mm in corresponding optical photo.

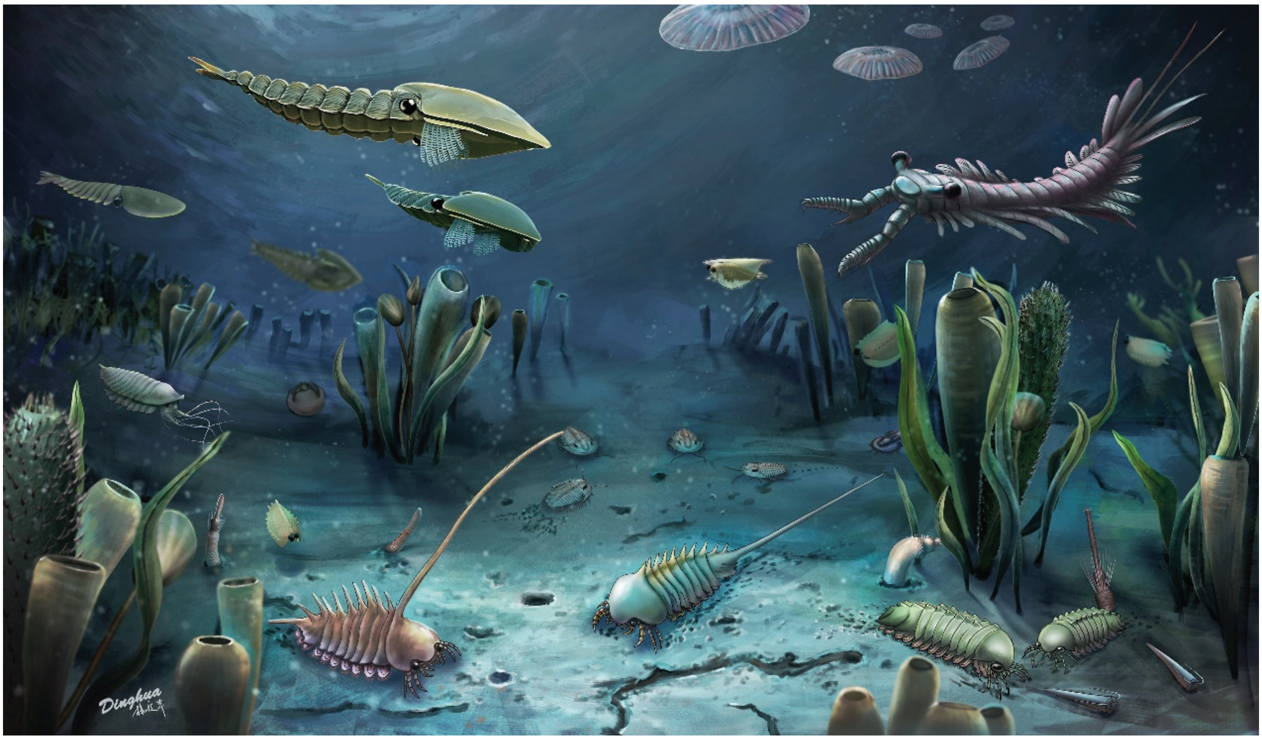

Supplementary Fig. 7. Life on the platform margin of the Miaolingian sea, North China. Based on data from the Linyi Lagerstätte. Drawing by Dinghua Yang.

Reference:

1. Aria C, Caron J-B. Mandibulate convergence in an armoured Cambrian stem chelicerate. *BMC Evol Biol* 2017; 17: 1–20.

2. Aria C, Caron J-B. A middle Cambrian arthropod with chelicerae and proto-book gills. *Nature* 2019; 573: 586–89.

3. Conway Morris S. Robison R A. More soft-bodied animals and algae from the Middle Cambrian of Utah and British Columbia. *University of Kansas, Paleontological Contributions* 1988; 122: 1–48.

4. Heymons R. Die Entwicklungsgeschichte der Scolopender. *Zoologica* 1901; 13: 1–244.

5. Lankester E R. The structure and classification of Arthropoda. *Quarterly Journal of Microscopical Science* 1904; 47: 523–82.

6. Lerosey-Aubril R, Kimmig J, Pates S, Skabelund J, Weug A, Ortega-Hernández J. New exceptionally-preserved panarthropods from the Drumian Wheeler Konservat-Lagerstätte of the House Range of Utah. *Pap Palaeontol* 2020a; 6: 501–31.

7. Lerosey-Aubril R, Skabelund J, Ortega-Hernández J. Revision of the mollisoniid chelicerate (?) *Thelxiope*, with a new species from the middle Cambrian Wheeler Formation of Utah. *PeerJ* 2020b; 8: e8879.

8. Ortega-Hernández J. Making sense of 'lower' and 'upper' stem-group Euarthropoda, with comments on the strict use of the name Arthropoda von Siebold, 1848. *Biol Rev* 2016; 91: 255–273.

9. Simonetta A M, Delle Cave L. The Cambrian non-trilobite arthropods from the Burgess Shale of British Columbia. A study of their comparative morphology, taxonomy and evolutionary significance. *Palaeontogr Ital* 1975; 69: 1–37.

10. Van Roy P, Orr P J, Botting J P, Muir L A, Vinther J, Lefebvre B, El Hariri K, Briggs D E G. Ordovician faunas of Burgess Shale type. *Nature* 2010; 465: 215–218.

11. Walcott C D. Cambrian geology and paleontology. Ⅱ. Cambrian faunas of China. *Smithsonian Miscellaneous Collections* 1911; 7: 69–108.

12. Yuan JL, Li Y and Mu XN *et al*. *Trilobite fauna of the Changhia Formation (Cambrian Series 3) from Shandong and Adjacent Area, North China. Part 1 and Part 2* (in Chinese). Beijing: Science Press, 2012, 1–757.
